# Reasons for missed chemotherapy appointments in retinoblastoma patients undergoing chemotherapy: A report from a Tertiary Care Hospital from India

**DOI:** 10.1002/cnr2.1279

**Published:** 2020-09-03

**Authors:** Aditya Kumar Gupta, Jagdish Prasad Meena, Rachna Seth

**Affiliations:** ^1^ Division of Pediatric Oncology, Department of Pediatrics All India Institute of Medical Sciences New Delhi Delhi India

**Keywords:** delayed chemotherapy, missed appointments, retinoblastoma

## Abstract

**Background:**

Delays in chemotherapy due to missed‐appointments can lead to sub‐optimal outcomes in any cancer. Missed appointments or delayed follow up are an important concern in the treatment of pediatric cancers as it compromises the patient's health and overall outcomes.

**Aim:**

This study was conducted to understand the reasons responsible for missed‐appointments in Retinoblastoma (RB) patients scheduled for daycare based chemotherapy.

**Methods:**

We prospectively recorded the causes for missed‐appointments in RB patients from February 2018 to September 2018. A delay of more than 48‐hours from the pre‐scheduled date of chemotherapy was categorized as a “missed‐appointment.”

**Results:**

Out of 870 scheduled visits of patients with RB for chemotherapy to our center, there were 122 (14%) instances of missed‐appointments during the study period. There were 40 instances (4.6%) where the patient had missed‐appointments (possibly avoidable reasons). These 40 instances occurred in 33 patients who had a median age of 29 months (IQR 22.5‐51.5 m) with 22 males. Six patients lived within 100 km of the treating center, 12 lived between 100 and 500 km, and 15 patients lived beyond 500 km. The median length of delay was 13.75 days (IQR‐7‐20.75 days). Twenty‐seven patients used a train as a means of transport, and 10 used the state‐bus. The main cause of delay was the illness of other family members (52.5%) followed by financial issues (27.5%), transport‐related problems (10%), and absence of an adult to accompany (10%).

**Conclusion:**

Causes for missed‐appointments for chemotherapy in RB patients were multifactorial and included the illness of other family members, financial issues, distance/transport‐related problems, and no caregiver to accompany. The future study with a large sample size with a multicenter design is needed to confirm the results of the current study and to know the deficiency in the improvement of the follow‐up RB patients.

**Abbreviation**
IQR: interquartile‐range
km: kilometer
LMIC: low‐middle‐income country
NGO: non‐government organization
RB: retinoblastoma
SD: standard deviation

## 1 INTRODUCTION

Adherence to treatment is defined as the extent to which a person's behavior corresponds with agreed recommendations from a health care provider.^1^ Abandonment of therapy in Oncology is defined as either missing at least four consecutive weeks of scheduled treatment or starting therapy after a cancer diagnosis. It is a major cause of adverse outcomes for pediatric cancers in developing countries.^2^ Whereas abandonment lies at the end of the spectrum of therapy non‐compliance, more general non‐adherence to treatment such as missing appointments and delays in follow up can also reduce the effectiveness of therapy. It also predicts a greater risk of abandonment of care.^3^

Missed appointments or delayed follow up are an important concern in the treatment of pediatric cancers as it compromises the patient's health and overall outcomes. Non‐adherence can particularly affect survival in cancer, where intense dose chemotherapy plays an important role. Positive influences which have known to increase adherence to scheduled appointments in chemotherapy are a positive family relationship and open communication in case of adolescent patients.^3^

The diagnosis of childhood cancer impacts the family in many ways. Many families suffer from unemployment and debts, and it is difficult for them to meet the transportation and medication expenses and the care of the remaining family members. This often leads to chronic poverty in countries where the government aid for treatment is lacking.^4^

Missed appointments and delays in follow up could be the first cry of help that could be indicative of unaddressed barriers to treatment. Systematic documentation of missed appointments has been shown to improve ways in which adherence could be increased. Preventive strategies are better in the overall improvement of outcomes compared to those after the abandonment.^5^

About 40% to 70% of children with retinoblastoma in the developing countries die, compared with 3% to 5% in the developed nations.^6^ The management of retinoblastoma involves multi‐specialty care and involves the modalities of chemotherapy, radiotherapy, and surgery. Chemotherapy is used for extraocular tumors to make them amenable to resection and is also used in the adjuvant setting in the presence of high‐risk features in intraocular tumors. The chemotherapy is administered at an interval of three to 4 weeks. Abandonment of therapy is the main cause of treatment failure in children of low‐middle‐income countries (LMIC), apparently because of limited resources and perceived stigma of cancer or loss of an eye. Delays and poor follow‐up is associated with sub‐optimal outcomes.^7^

Chemotherapy delays as a result of missing appointments in RB can be a result of avoidable or non‐avoidable reasons. Unavoidable reasons include causes like delayed recovery from a surgical procedure or the previous round of chemotherapy. Avoidable reasons for the delay could be many and are the potential ones that could be addressed. The present study was done to investigate the causes responsible for missing appointments and chemotherapy delays in patients undergoing chemotherapy for RB.

## 2 MATERIAL AND METHODS

### 2.1 Patient selection criteria

This prospective observational study conducted from February 2018 to September 2018 at the Division of Pediatric Oncology, of the Department of Pediatrics in All India Institute of Medical Sciences (AIIMS), New Delhi, India. The permission for the same was taken from the Institute Ethics Committee (IEC‐713/29.12.2017, RP‐23/2018). The AIIMS is a tertiary care referral center for the public sector in the country. The hospital is one of the major centers catering to RB patients in the country. We enrolled children age <18 years on chemotherapy for RB who had missed appointment and delayed for follow up >48 hours. Both the treatment appointments and routine follow‐ups missed by these patients were recorded. The exclusion criteria were: missed appointments due to infection or illness after the previous chemotherapy cycle or surgery; missed appointment due to delayed count recovery or derangement in pre‐chemotherapy investigations; missed appointment due to delay in getting the investigations done in the hospital. Children were included if the parent/legally authorized representative (LAR) signed informed consent.

### 2.2 Objectives

The objective of the study was to determine the factors that contribute to missed appointments and delayed follow‐up in RB patients on chemotherapy. The outcome variables were: median delays of chemotherapy (days); reasons for missed appointments and delays after which phase of chemotherapy.

### 2.3 Definitions

#### 2.3.1 Missed‐appointment

A delay of more than 48‐hours from the pre‐scheduled date of chemotherapy was categorized as a “missed‐appointment.”

### 2.4 Statistical analysis

The categorical variables were presented as frequency (percentage). The continuous data were presented as mean ± SD for normally distributed data, and as median with interquartile range (IQR) for skewed data. Statistical analysis was carried out using the STATA software (STATA version 14, for Windows 10).

## 3 RESULTS

**FIGURE 1:**
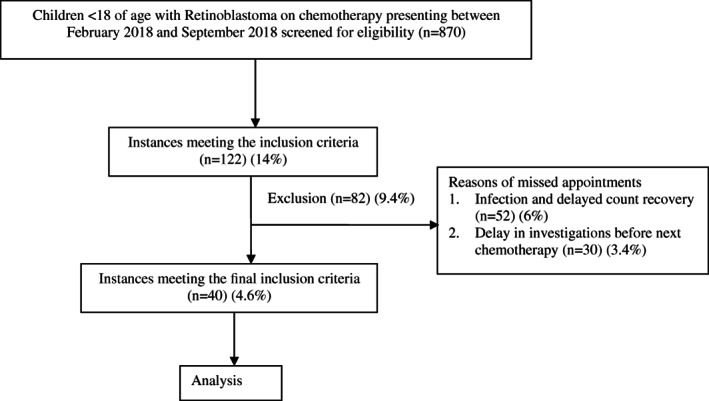
Flow diagram of study

Out of a total of 870 scheduled visits during the study period to the Pediatric Oncology daycare for RB chemotherapy, there were 122 instances (14%) where the patient was delayed by more than 48 hours. Of these, 52 instances (6%) were due to the patient being neutropenic or due to having an infection or with delayed counts recovery that precluded chemotherapy. In 30 cases (3.4%), the patient would have come on time, but there was a delay in getting the pre‐chemotherapy laboratory tests done (Figure 1). The remaining instances of delays (n = 40, 4.6%) were unanticipated and these 40 instances of delay happened in 33 patients.

The median age of these 33 patients was 29 months (IOR 22.5‐51.5 months). Sixteen patients had an extraocular disease and in 11 patients, the disease was bilateral. Thirteen patients were on standard doses of chemotherapy, whereas in 20, the doses were higher for either an extraocular presentation or sub‐optimal response to previous chemotherapy cycles (Table 1).

**TABLE 1:** Demographics and patient characteristics who missed appointments for retinoblastoma chemotherapy (40 missed appointments in 33 patients)

Demographics	n = 33	95% CI
Males: Females	22:11	Male: 0.49 to 0.83 Female: 0.16 to 0.50
Median age (mo) IQR	29 (22.5‐51.5)	‐
Unilateral RB: Bilateral RB	22:11	Unilateral: 0.49‐0.83 Bilateral: 0.16‐0.50
Intraocular RB: Extraocular RB	17:16	Intraocular: 0.33‐0.69 Extraocular: 0.30‐0.66
Standard Chemotherapy: Intensified Chemotherapy	13:20	Standard: 0.21‐0.56 Intensified: 0.43‐0.78
**Distance from treating centre**		
<100 km	6 (18.1%)	0.04 to 0.32
100 to 500 km	12 (36.4%)	0.19 to 0.53
>500 km	15 (45.5%)	0.27 to 0.63
Non cemented house: Cemented house:	24:9	0.57 to 0.88
No education: Some education (of any one parent)	11:22	No education: 0.16 to 0.50 Some education: 0.49 to 0.83
Average income per household per month in INR	9560/‐	5115.51 to 14 005.69
Average number of members per household	8	6.53 to 9.52
**Local place of stay (in Delhi)**		
Relative's residence	9 (27.3%)	
NGO supported accommodation	9 (27.3%)	‐
Rented accommodation	7 (21.2%)	
No place to stay	8 (24.2%)	
Returned to original place of residence after chemotherapy	27 (81.8%)	0.04 to 0.32
Previous radiation therapy	7 (21.2%)	0.61 to 0.91
Previous enucleation	13 (39.4%)	0.43 to 0.78
Previous defaulters	13 (39.4%)	0.43 to 0.78
Co‐morbidities in the patient	3 (9%)	0.80 to 1.01
Chronic‐illness in any family member	6 (18%)	0.67 to 0.95
**Mode of transport (n = 40)**		
Train	27 (67.5%)	
State Bus	10 (25%)	‐
Others	3 (7.5 %)	

Abbreviations: CI, confidence interval; IQR, interquartile range; NGO, non‐govt organization; RB, retinoblastoma.

Six patients lived within 100 km of the treating center, 12 lived between 100 and 500 km, and 15 patients lived beyond 500 km. Twenty‐five patients lived in ill‐constructed (thatched house/hut). In 11 cases, both parents had never been to school, whereas for the rest, at least one of the parents had some form of schooling (only two families had a graduate as a parent). The families' average monthly income was 9560 Indian National Rupees (INR; approx. UDS 135). In six families, another family member was suffering from a chronic illness. The average numbers of family members were eight per household. Locally at the primary treating center, nine patients stayed at a relative's house, nine had accommodation provided by the Govt/Non‐Govt organization (NGO), whereas eight families had no place to stay and they put up at the roadside /open public spaces. Twenty‐seven of the 33 patients returned to their original place after each cycle of chemotherapy.

Out of the 33 patients, 7 patients had the previous radiotherapy, and 13 patients had an enucleation. Thirteen patients had been previous defaulters, even before the first enrolment in the study. After the first enrolment in the study, six patients missed appointments again during the study period. Three patients had co‐morbidities [two‐developmental delay, one‐chronic suppurative otitis media (CSOM)]. Twenty‐seven families used trains as a means of transport, and 10 used the state‐bus.

The median number of days of the delay of chemotherapy was 13.75 days (IQR 7‐20.75 days). The main cause of delay was an illness of other family members (52.5%) followed by financial issues (27.5%), transport‐related problems (10%), and absence of an adult to accompany (10%). More than 50% of patients had missed appointments during the first 4 cycles of chemotherapy (Table 2).

**TABLE 2:** Details of missed chemotherapy appointments in retinoblastoma patients undergoing chemotherapy (n = 40)

Variables	n = 40	95% CI
Median delay (d) IQR	13.75 (7‐20.75)	–
**Reasons for the delay**		
Illness of family member	21 (52.5%)	0.33 to 0.69
Financial issues	11 (27.5%)	0.04 to 0.32
Transport	4 (10%)	0.003 to 0.23
No care giver in family	4 (10%)	0.003 to 0.238
**Delay after chemotherapy cycle**		
First	5 (12.5%)	0.003 to 0.23
Second	5 (12.5%)	0.02 to 0.28
Third	6 (15%)	0.004 to 0.24
Fourth	5 (12.5%)	0.013 to 0.19
Fifth	3 (7.5%)	0.013 to 0.19
Sixth	3 (7.5%)	0.013 to 0.19
Seventh	2 (5%)	0.03 to 0.15
Eighth	2 (5%)	0.03 to 0.15
Ninth	3 (7.5%)	0.02 to 0.15
Tenth	2 (5%)	0.03 to 0.09
Eleventh	3 (7.5%)	0.01 to 0.19
Twelfth	1 (2.5%)	0.03 to 0.09

Abbreviations: CI, confidence interval; IQR, interquartile range.

## 4 DISCUSSION

In the treatment of any malignancy, it is crucial to adhere to schedules and dates of chemotherapy. Delays in chemotherapy in adult cancers such as colon cancers are known to be associated with poor outcomes.^8^ The RB involves multi‐modality super‐specialty care for the best outcomes. The primary purposes of the application of systemic therapy are to reduce the tumor size for local treatment (chemo reduction) or to reduce the risk of metastasis after enucleation surgery (adjuvant therapy).^9^

In the present study, we found that in 14% of instances, there was a delay of more than 48 hours for reporting for chemotherapy. Approximately 5% of delays (n = 40) in 33 patients of RB were due to reasons which could have been avoided.

In a review published by Ambroggi et al^10^ travel burden was an important factor affecting access to cancer diagnosis and treatment and could hinder the achievement of quality care for cancer patients. The implications of increasing travel distance can be profound. It has been reported that even a small increase in distance can result in a substantial barrier for optimal cancer treatment.^11^ In our study, about half of these patients had to travel more than 500 km to get the chemotherapy administered.

The main cause of delayed chemotherapy administration was the illness of the other family members (52.5%), followed by financial issues in this study. In about 20% of the families, another family member was suffering from a chronic illness.

In a country like India where the burden of RB is high, it is important to establish a grid of multi‐specialty cancer centers so that facilities for early diagnosis and optimal treatment are accessible to the majority of the population. The care of RB often requires the patient to reach urgently to the treatment centers. The treatment center should ideally be near the permanent place of residence of the patient so that the travel burden is minimized and social support is available to the patient. In our cohort, 81% of patients returned home after each chemotherapy cycle. Understandably, these RB families' may have other social and economic demands to attend, which lures them to return home after the 2 days of chemotherapy. This could be risky because it leads to the patient becoming more distant from the primary treating center, and in case of an emergency resulting from the chemotherapy, the patient may only be left with sub‐optimal health care access.

In our cohort, most of them had a non‐cemented accommodation, also called a “kaccha house” in India. Whereas in 11 families, both parents had no exposure to education, in the case of two, at least one parent was a graduate. Even in the ones where either parent has some form of schooling, it was below the level of matriculation. The average income of the household was approx. INR 9560 (approx. 135 USD) per month. The socioeconomic and educational lag of these families is a major hindrance to health‐seeking behavior.

Poor accommodation may result in the patient with RB becoming vulnerable to infections and malnutrition. Poor education is a deterrent in the family's understanding of the complexities associated with the care of a RB child. Financial insecurity in the absence of social security schemes can make the RB child vulnerable to delays and abandonment of treatment. Lower socioeconomic status is known to be associated with poor overall survival in cancers.^12^

Socioeconomic differences in cancer survival are well known. Apart from the fact that patient with poor socioeconomic status have a delayed presentation and diagnosis, evidence of differential treatment between social groups.^13^

The average number of family members per household was eight in our cohort. A large family may be a boon in certain situations where it leads to a better economic and social support system. In cases where the family's socioeconomic and educational backbone is fragile, more‐family members would mean more liability for the head of the household. It carries the risk of the income being divided, and the time and attention that the child with RB is entitled to are often distributed amongst others. The treatment of the child with RB is emotionally, financially, and socially taxing. A robust way for tackling this problem would be to provide state‐sponsored support to all children diagnosed with RB.

Eight out of the 33 families had no place to stay in the city when they arrived for chemotherapy. Seven stayed in rented accommodation, and nine families put up with some relatives. Absence of a reassuring place of stay even during the chemotherapy can deter families from optimal healthcare‐seeking behavior. Lack of proper accommodation exposes the RB child undergoing chemotherapy to hazards of weather, pollution, and unhealthy food. To facilitate the treatment of RB patients, more support from Government and NGO is desired so that all patients who leave their place of residence and venture out for treatment have a humane and decent accommodation.

Loh et al published a review about cancer supportive care in Singapore, and they suggested that core working groups across tertiary and community health care providers and institutions, as well as collaboration with governmental and volunteer welfare organizations, are necessary to advance the field of supportive care for the benefit of cancer patients and their families.^14^

In our cohort, even at the initial enrolment, 13 patients had missed appointments earlier. This probably indicates a persistent stressor in the family and its macroenvironment and microenvironment that deters optimal healthcare‐seeking behavior. A study done on lung cancer patients found that 35% of patients were intermittent defaulters. The default may result in delaying the appropriate treatment, which affects outcomes and even mortality. Reasons which have been noted for default in various studies include symptom duration or resolution, illness, long waiting periods, forgotten appointments, work commitment, illness, hospital administrative error, and transport problems.^15^

In a country with the second‐highest population, traveling to reach the primary treatment center may be a herculean task. Most of the patients in our study used trains as a means of transport. In India, the trains are often overcrowded, and it is often difficult to get travel reservations on a given date. Transport related issues were related to missing appointments in four patients. Guidry et al published that cancer patients may opt to forgo needed care in the absence of available and affordable means of transportation to treatment facilities.^16^

Apart from other family members' illness and transport‐related reasons, the other reasons responsible were lack of finances and the absence of a caregiver to accompany the child. For the parent of the RB child who may be the only earning member in the family, it may often be difficult to arrange for a person to accompany the child. If the parent has to accompany, this would mean losing on days of work, and this precarious balance may tilt on the side of delay due to the adverse circumstances.

Affordable and equitable cancer care in India remains a challenge as the state expenditure as per a review published in 2014 and was below 10 USD per patient, and overall spending on healthcare is approximately 1% of the GDP. Out of pocket expenditures pose the danger of negatively affecting the socio‐economic milieu of the family and forthcoming generations. Social security schemes will have a big impact on the optimizing of care for these patients.^17^

The strength of this study was the prospective enrolment of RB patients on chemotherapy for the understanding of reasons for missed‐appointments. *Limitations*: This study primarily focuses on reasons for missed‐appointments for chemotherapy in RB patients and the survival, and the long term outcomes were not looking for. We did not record the data of patients who did not turn up for the treatment at all, and we also did not take a comparative group in this study. The small sample size was also a limitation of the study.

## 5 CONCLUSION

Causes for missed‐appointments for chemotherapy in RB patients were multifactorial and included the illness of the other family members, financial issues, distance/transport, and no caregiver. The future study with a large sample size with a multicenter design is needed to confirm the results of the current study and to know the deficiency in the improvement of the follow‐up of patients with RB.

## CONFLICT OF INTEREST

The authors declare no conflicts of interest.

## AUTHOR CONTRIBUTIONS

All authors had full access to the data in the study and take responsibility for the integrity of the data and the accuracy of the data analysis.

*Conceptualization*: A.K.G.; *Methodology*: A.K.G.; *Investigation*: A.K.G.; *Formal Analysis*: A.K.G. and J.P.M.; *Resources*: A.K.G.; *Writing*: A.K.G. and J.P.M.; *Writing ‐ Review & Editing*: A.K.G., J.P.M., and R.S.; *Visualization*: R.S.; *Supervision*: R.S.; *Funding Acquisition*: None.

## ETHICS STATEMENT

The ethics clearance was obtained from the Institute Ethics Committee (IEC‐713/29.12.2017, RP‐23/2018), All India Institute of Medical Sciences (AIIMS), New Delhi, India. Children were included if the parent/ legally authorized representative (LAR) signed informed consent.

## Data Availability

The data that support the findings of this study are available from the corresponding author upon reasonable request.
